# Evaluation of Mast Cells in Oral Potentially Malignant Disorders and Oral Squamous Cell Carcinoma

**DOI:** 10.1155/2021/5609563

**Published:** 2021-08-27

**Authors:** Ashish Shrestha, Shashi Keshwar, Toniya Raut

**Affiliations:** Department of Oral Pathology, BP Koirala Institute of Health Sciences, Dharan, Nepal

## Abstract

**Background:**

Oral squamous cell carcinoma (OSCC) and oral potentially malignant disorders (OPMDs) are epithelial-derived pathologies which share inflammation as a common initial pathogenic-inducing state. Mast cell is a key immune modulating cell which is primarily involved in initiation and propagation of inflammation. The role of mast cell in OPMDs and OSCC has been an established fact; however, its definitive pathogenic correlation is still under study. The objective of the study was to evaluate the number of mast cell in OPMDs and OSCC using special stain correlating its probable role as a promoter or retarder of OSCC.

**Materials and Methods:**

Forty-five archival histopathologically confirmed cases each of OPMD and OSCC were studied for mast cells using toluidine blue and Alcian blue-safranin stain and compared with 10 normal oral mucosal tissues. Comparisons between the mast cells count was also performed between the two special stains.

**Results:**

Among 100 cases, 67% were males and 33% were females. The mean age was 41.68 ± 13.39; 55.06 ± 12.55; and 18.4 ± 2.54 years for OPMDs, OSCC, and normal, respectively. A statistically significant increase in mast cells among OPMDs (9.88 ± 7.9) and OSCC (6.711 ± 3.94) was observed compared to normal oral mucosa. The mast cell count reduced among OSCC in comparison to OPMDs which was significant as well. The mean mast cell count for Alcian blue-safranin stain was higher than toluidine blue stain.

**Conclusion:**

There is a decrease in mean mast cell count from OPMDs to OSCC which is suggestive of protective role of mast cell. Proper quantification of mast cells using specific stains can guide to define prognosis in oral potentially malignant disorders and oral cancer.

## 1. Introduction

Mast cells form an immune system tranche that plays an important role in host defense. They are present in vascularized tissues, particularly in connective tissues and mucosal surfaces [1]. Variable evidence exists in context to the role of mast cells as a metastasis promoter in some tumor and a protective rejecter in others [2]. The propensity of mast cells to concentrate in inflammatory and neoplastic focus around blood vessels has been established which later showed that they accumulate around tumors before tumor-related angiogenesis starts [3].

Inflammation is a sword with double cutting edge, responsible for both defense and protection against the carcinogenic agent such as tobacco, but at the same time, it results into tissue destruction. Likewise, the pathogenesis of oral potentially malignant disorders (OPMDs) and oral squamous cell carcinoma (OSCC) begins with the inflammatory response, mediated by immune cells such as mast cells, neutrophils, lymphocytes, macrophages, and others [4].

Special stains such as toluidine blue and Alcian blue-safranin stain are specific to mast cells which otherwise are difficult to identify with routine hematoxylin and eosin stain. Toluidine blue is a simple metachromatic thiazine dye that is most widely used for mast cell identification [4]. The metachromatic property of toluidine blue imparts purple or reddish-blue color to the sulphated granules (mostly heparin) of the mast cell, depending on the degree of polymerization of the dye, making it easier to recognize [5]. The mast cell containing both sulphated (heparin) and carboxylated (histamine) mucopolysaccaride and sialomucin in the cytoplasm can be stained by the Alcian blue-safranin O stain [6].

Keeping in context the staining property of mast cells with different stains and associated immune response in pathologies of oral cavity, this study was designed for comparative quantification of mast cell in potentially malignant oral disorders and oral squamous cell carcinoma with normal oral mucosa.

## 2. Materials and Methods

A retrospective study was conducted including histopathologically confirmed 45 cases each of oral potentially malignant disorders and oral squamous cell carcinoma and ten cases of normal oral mucosa from the archive of the Department of Oral Pathology. Oral potentially malignant disorders included 15 cases each of oral submucous fibrosis, oral lichen planus, and oral epithelial dysplasia. The oral squamous cell carcinoma cases comprised 23 cases of well-differentiated and 22 cases of moderately differentiated squamous cells carcinoma. The cases for normal oral mucosa were obtained as mucosa obtained during extraction of impacted molars. Ethical clearance was obtained from the Institutional Review Committee of the Institute.

The routine stained slides of the cases were reviewed from the archives of the department for confirmation and labeling. Subsequent paraffin-embedded tissue blocks were selected and sectioned into 3 *μ*m each and stained with toluidine blue and Alcian blue-safranin stains separately following standard staining protocol of the department.

Round-to-polygonal cells with a centrally placed nucleus with a purple-colored granular cytoplasm were considered positive for toluidine stains, and cells with a blue- or red-colored granular cytoplasm for Alcian blue-safranin stain were considered as positive identification of mast cells.

Random non-overlapping five fields were selected by an oral pathologist who was blinded regarding the diagnosis of the cases. Photomicrographs of those five fields were taken in high-power magnification using the VEGA 5.0 Camera (PixelPro 3.0 Software; Labomed, USA) attached with a research microscope. The same photographs were evaluated three times for identification and counting of the mast cells to avoid observational bias. The average of the number of mast cell count during the three episodes was considered for statistical analysis. The unpaired *t*-test was used to compare the mean number of mast cells among study groups, keeping the *p* value less than 0.05 as statistically significant.

## 3. Results

The 100 study samples comprised 67% (*n* = 67) males and 33% (*n* = 33) females. The mean age of cases of OPMD was 41.68 ± 13.39 years; OSCC 55.06 ± 12.55 years; and 18.4 ± 2.54 years of normal oral epithelium.

The mean mast cell count was the lowest among the normal group and the highest among the epithelial dysplasia group. Mast cell count using toluidine blue and Alcian blue-safranin stains for the normal group was 4.88 ± 1.80 and 5.6 ± 1.94, respectively, whereas for the epithelial dysplasia group, it was 12.55 ± 11.57 and 12.34 ± 8.9, respectively (Table 1).

**Figure 1:**
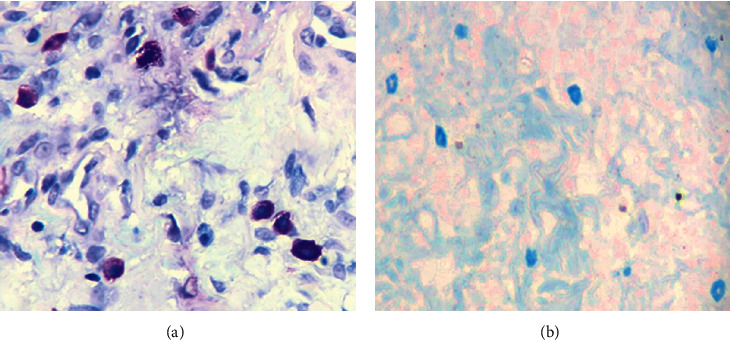
Toluidine blue (a) and Alcian blue-safranin (b) staining of mast cells in OSCC (40x).

The mast cell count increased in both OPMDs and OSCC compared to the normal oral mucosa, whereas it was the highest among OPMDs followed by OSCC. Comparison of mean mast cell count was performed among normal and OPMDs, normal and OSCC, and OPMDs and OSCC for both toluidine blue and Alcian blue-safranin stain (Figure 1(a) and 1(b)). All the comparisons were statistically significant except the comparison between OPMDs and OSCC using Alcian blue-safranin stain (Table 2).

Both toluidine blue and Alcian blue-safranin stains were compared for their ability to stain mast cell between which Alcian blue-safranin stain comparatively stained more mast cells among OSCC, OPMD, and normal tissues. A statistically significant difference between the stains was noted only among cases of oral squamous cell carcinoma (Table 3).

## 4. Discussion

Mast cell is a bone-marrow-derived tissue-homing leukocyte which has an active role in a wide biological spectrum of inflammation, immune modulation, angiogenesis, and many more. It is unique and histologically demonstrable with its metachromatic granules containing heparin, protease, and cytokines [7]. Apart from its degranulation in allergic reaction, it is associated with ‘piece meal degranulation,' i.e., a selective pathway of cell secretion which aggravates progression of oral potentially malignant disorders (OPMDs) to oral squamous cell carcinoma (OSCC) through angiogenic switch. The angiogenic factor released by mast cell provides survival and progressive ability [8].

The role of mast cell has been documented in a wide range of OPMDs including oral leukoplakia, oral lichen planus, and oral submucous fibrosis [9–12]. The present study showed an increased level of mast cells in OPMDs compared to normal mucosa which was statistically significant. Interleukin-1 released from degranulated mast cell has been associated with epithelial proliferation in oral leukoplakia. Histamine increases the mucosal permeability and facilitates the accessibility of the irritants to the underlying connective tissue increasing the probability of epithelial dysplasia [13]. Major histocompatibility class I-associated CD8-cell activation in oral lichen planus results in mast cell degranulation through its cytotoxic effect. The degranulated mast cell, thus, releases TNF-alpha which further releases matrix metalloproteinase-9, breaking the integrity of basement membrane and increasing the chronicity of oral lichen planus. Apart from this; TNF-alpha also operates a cyclic pathway of continuous release and degranulation of mast cell through RANTES (regulated on activation of normal T cell expressed and secreted) [9].

The role of mast cell degranulated chemokines has also been clearly mentioned for the initiation and progression of oral submucous fibrosis. Mast cells get compiled in OSMF in response to the areca nut-associated irritation. The release of histamine, serotonin, chymase, and tryptase together contribute for early-stage changes such as burning sensation, itching, stomatitis, and increased salivation [10, 13]. Increased fibroblast proliferation with upregulation of procollagen mRNA is also implicated to mast cell-released tryptase [14].

In the presence of a carcinogenic environment, a continuous emerging oncogenic signal evokes normal cell conversion to OPMDs and OPMDs to OSCC. Activation and recruitment of mucosal or connective tissue mast cells are in response of chronic inflammation either in OPMDs or in OSCC [8, 11].

In the present study, despite generalized increase in the level of mast cells in both OSCC and OPMDs compared to normal, the level was reduced in OSCC than in OPMDs. A similar observation was seen in the study conducted by Oliviera et al., where they concluded that once the tumor microenviroment was established, there was high probability for failure of migration of mast cells at the site of tumor [15]. A few hypotheses have been formulated by the author in relevance of mast cell migration which state that failure of mast cell infiltration might be either due to reduced chemotactic factors to attract mast cells or downregulation of the c-kit activation pathway which are the prerequisites for mast cell migration [15]. A similar finding was observed by Shreya et al. [4] while evaluating mast cell in OPMDs and OSCC using toluidine blue stain. On contrary to the finding of the present study, Iamaroon et al. [16] and Michailidou et al. [12] found a significant increase in the number of mast cells in cases of OSCC compared to OPMDs. These findings were correlated to the probable angiogenic switch that might occur in the early stage of malignant transformation and emphasized on to the role of mast cell in progression of normal tissue to dysplastic leading to OSCC.

The association of mast cell with pathogenesis of OSCC has been through the controversial dual role. A group of experts believe its assistance to progress the tumor through angiogenesis and neovascularization; on contrary, the other group of experts support the cytotoxic function of the mast cell that suppresses the tumor growth potentials [4, 7, 15, 17]. This dual role has been recapitulated as the cytotoxic effect of mast cells is dominated in the initial stage of tumor infiltration; however, once the tumor is established, the altered tumor microenvironment suppress the mast cell infiltration by various mechanisms with which its cytotoxic effect is suppressed favoring its angiogenic potential for tumor growth. This probable mechanism is potentiated with the finding that the cytotoxic effect of mast cell is actively associated with a mast cell-to-tumor ratio of more than 20 : 1 that reverses when the ratio changes from 10 : 1 to 1 : 100 [18, 19].

Special stains such as toluidine blue and Alcian blue-safranin stain are specific to identify mast cells. Toluidine blue is a simple metachromatic thiazine dye that is most widely used for mast cell identification [4]. The metachromatic property of toluidine blue imparts a purple or reddish-blue color to the sulphated granules (mostly heparin) of the mast cell, depending on the degree of polymerization of the dye, making it easier to recognize [5]. The mast cell containing both sulphated (heparin) and carboxylated (histamine) mucopolysaccaride and sialomucin present in its cytoplasm can be stained by the Alcian blue-safranin O stain [6].

The present study has also evaluated the staining potential of two metachromatic dyes, toluidine blue and Alcian blue-safranin stain. The mean mast cell count from Alcian blue-safranin stain was higher in all the groups in comparison with toluidine blue staining which could be probably due to the propensity of Alcian blue for both sulphated heparin as Alcian blue coloration and histamine as a combination of Alcian blue and safranin stain, whereas in toluidine blue, the metachromasia is mainly directed for heparin-contained granules [20, 21].

There are many diagnostic and prognostic immunohistochemical markers for OPMDs and OSCC; however, due to economic constrain and requirement of an advanced laboratory setup for molecular study, patients with low economic status can hardly afford where an economically reasonable diagnostic modality such as histochemical stains would always be an option to choose.

Despite that the study could not tie-in with the gradual increase in mast cell progressively from normal to OPMDs to OSCC, there is no uncertainty to admit that there is a definitive role of immune mast cell behind the pathogenesis of malignant and potentially malignant disorders. As mast cells are preloaded with numerous intrinsic components, both with neoangiogenic and cytotoxic outcome, emphasis on its cytotoxic aspect would be an innovative aspect for management of malignant and potentially malignant disorders.

## 5. Conclusions

Mast cells play an important part in a wide range of physiological and pathological processes. They serve as immune system gatekeepers, responding to a variety of signaling pathways and, thereby, leading to carcinogenesis and metastasis. There is a decrease in mean mast cell count from OPMDs to OSCC which is suggestive of the protective role of mast cell. Proper quantification of mast cells using specific stains can guide to define prognosis in potentially malignant disorders and oral cancer.

## Figures and Tables

**Table 1:** Mast cell count using toluidine blue and Alcian blue stain among subgroups.

Group	Subgroup	Number	Mast cell count (mean ± SD)	
			Toluidine blue	Alcian blue-safranin
Normal	Normal	10	4.88 ± 1.80	5.6 ± 1.94

OPMDs	Lichen planus	15	9.50 ± 6.44	11.86 ± 8.01
	Oral submucous fibrosis	15	7.6 ± 3.28	7.05 ± 2.31
	Epithelial dysplasia	15	12.55 ± 11.57	12.34 ± 8.9

OSCC	Well-differentiated SCC	23	7.38 ± 4.40	11.31 ± 6.8
	Moderately differentiated SCC	22	6.00 ± 3.34	8.25 ± 4.6

**Table 2:** Intergroup comparison of mast cell using toluidine blue and Alcian blue-safranin stain.

Comparative group	Toluidine blue stain		Alcian blue-safranin stain	
	Mean ± SD	*p* value	Mean ± SD	*p* value
Normal	4.88 ± 1.80	0.00	5.6 ± 1.94	0.00
OPMDs	9.88 ± 7.96		10.42 ± 7.30	
Normal	4.88 ± 1.80	0.03	5.6 ± 1.94	0.00
OSCC	6.71 ± 3.94		9.81 ± 6.03	
OPMDs	9.88 ± 7.96	0.01	10.42 ± 7.30	0.66
OSCC	6.71 ± 3.94		9.81 ± 6.03	

**Table 3:** Comparison of mast cells between toluidine blue stain and Alcian blue-safranin stain.

Groups	Number	Toluidine blue	Alcian blue-safranin	*p* value
Normal	10	4.88 ± 1.80	5.6 ± 1.94	0.40
OPMDs	45	9.88 ± 7.96	10.42 ± 7.30	0.74
OSCC	45	6.71 ± 3.94	9.81 ± 6.03	0.00

## Data Availability

The data that support the findings of this study are available on request from the corresponding author.
